# RadD is a RecA-dependent accessory protein that accelerates DNA strand exchange

**DOI:** 10.1093/nar/gkac041

**Published:** 2022-02-12

**Authors:** Nina J Bonde, Zachary J Romero, Sindhu Chitteni-Pattu, Michael M Cox

**Affiliations:** Department of Biochemistry, University of Wisconsin-Madison, Madison, WI, USA; Department of Biochemistry, University of Wisconsin-Madison, Madison, WI, USA; Department of Biochemistry, University of Wisconsin-Madison, Madison, WI, USA; Department of Biochemistry, University of Wisconsin-Madison, Madison, WI, USA

## Abstract

In rapidly growing cells, with recombinational DNA repair required often and a new replication fork passing every 20 min, the pace of RecA-mediated DNA strand exchange is potentially much too slow for bacterial DNA metabolism. The enigmatic RadD protein, a putative SF2 family helicase, exhibits no independent helicase activity on branched DNAs. Instead, RadD greatly accelerates RecA-mediated DNA strand exchange, functioning only when RecA protein is present. The RadD reaction requires the RadD ATPase activity, does not require an interaction with SSB, and may disassemble RecA filaments as it functions. We present RadD as a new class of enzyme, an accessory protein that accelerates DNA strand exchange, possibly with a helicase-like action, in a reaction that is entirely RecA-dependent. RadD is thus a DNA strand exchange (recombination) synergist whose primary function is to coordinate closely with and accelerate the DNA strand exchange reactions promoted by the RecA recombinase. Multiple observations indicate a uniquely close coordination of RadD with RecA function.

## INTRODUCTION

**Figure 1.**
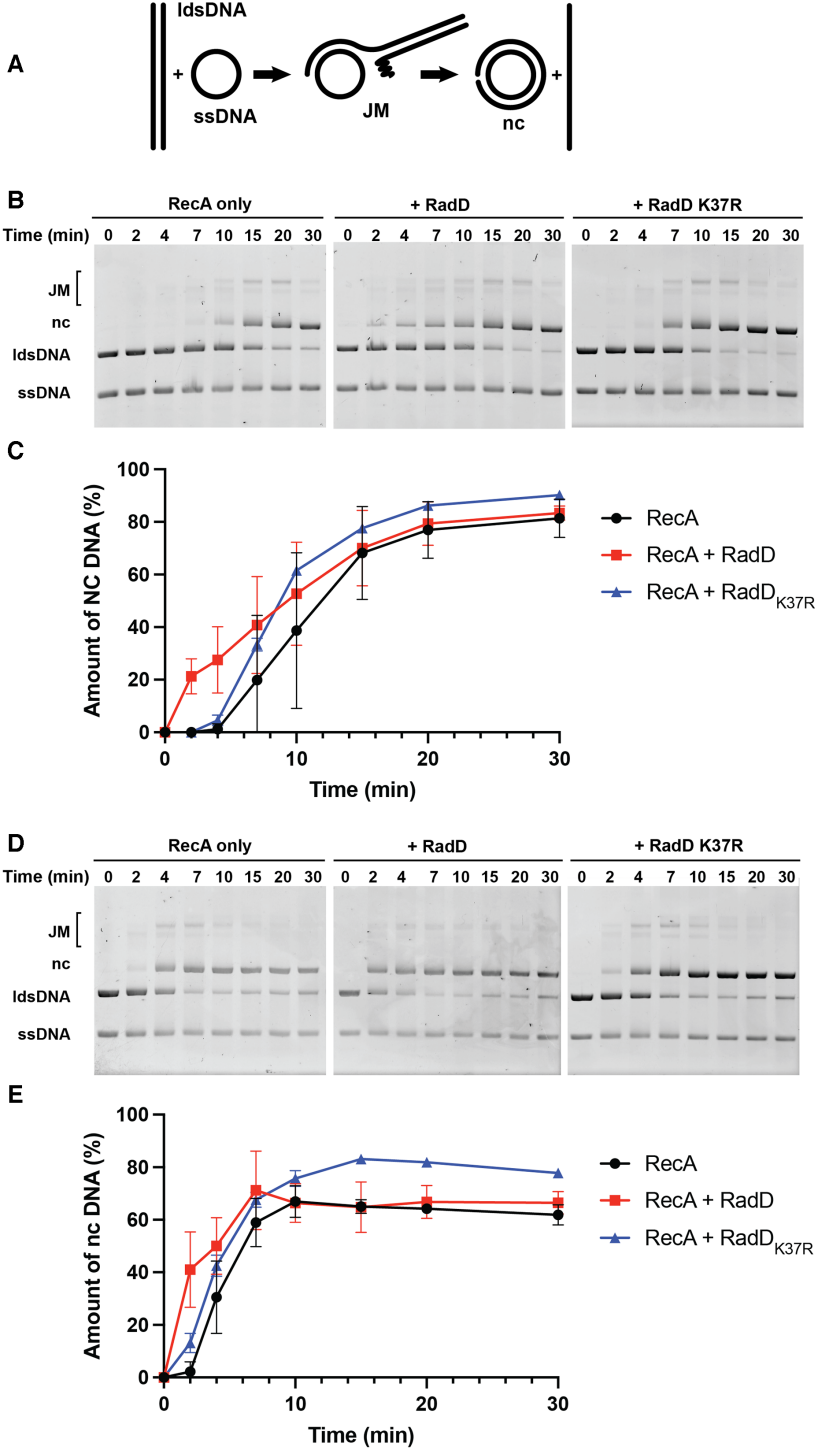
RadD stimulates RecA mediated strand exchange. (**A**) Reaction scheme. (**B**) Strand exchange reaction time courses in reactions lacking RadD or with RadD or RadD K37R added immediately after the dsDNA. Reactions contained 20 μM circular φX174 ssDNA (3.7 nM in molecules), 20 μM linear φX 174 dsDNA (1.86 nM in molecules), 3 μM RecA protein, 2.1 μM SSB, and 3.7 nM RadD protein (a 2:1 ratio of RadD molecules to linear dsDNA (ldsDNA) molecules). (**C**) Quantifications of nicked circular (nc) DNA from three independent strand exchange reactions shown in A with the average values plotted and standard deviations represented with error bars. (**D**) Strand exchange reactions carried out at the optimal 1 RecA per 3 nt cssDNA ratio in the absence or presence of RadD or RadD K37R, added immediately after the linear dsDNA. All components are at concentrations listed for panel B except that the RecA concentration is increased to 6.7 μM, a concentration sufficient to saturate the available ssDNA binding sites. (**E**) Quantifications of nc DNA from three independent strand exchange reactions shown in panel D with average values plotted and strand deviations represented.

In oxygenated *Escherichia coli* cells growing in rich media, the repair of stalled or collapsed replication forks occurs in nearly every replication cycle (1–7). Much of the repair involves recombinational DNA repair mediated by the RecA protein. RecA may help repair double strand breaks, fill in post-replication gaps with bypassed lesions, or facilitate fork regression when a fork has stalled (6,8–14). Each repair event must occur within a time interval that is framed by replication forks that may pass every 20 min. However, RecA-mediated DNA strand exchange, consisting of strand invasion followed by extension of the heteroduplex region via branch migration (Figure 1A), is surprisingly slow. The branch migration mediated by a RecA filament proceeds at only about 360 bp/min (15–17). With RecA typically responsible for only one step in a complex repair process, the time required for RecA-mediated extension of heteroduplex DNA after strand invasion seems to defy kinetic competence with respect to normal bacterial DNA metabolism.

Some proteins that have auxiliary roles in recombination can facilitate more rapid movement of the branched structures created by RecA protein. When strand exchange creates a Holliday junction, branch migration can be accelerated by the RuvA and RuvB proteins (18–21). The RuvAB proteins can also act as an anti-recombinase system, reversing RecA mediated strand exchange that has been halted by heterologous barriers (22). The RadA protein is an alternative system that can greatly stimulate RecA-mediated DNA strand exchange reactions *in vitro* (23). The RecG protein is responsible for the processing of many different branched structures at stalled replication forks and at recombination intermediates (24–28). RecG has an inhibitory effect on RecA-mediated DNA strand exchange, effectively reversing the reactions most likely to occur in post-replication gaps (27,28). RuvAB and RadA both promote their helicase and/or branch migration functions on appropriate branched DNA substrates in the absence of RecA (23,29). It is not yet clear how and when these activities are coordinated with RecA function, although both clearly play an important role in at least some repair contexts.

The ***radD*** gene (formerly *yejH*) encodes a polypeptide of 586 amino acid residues (including the N-terminal Met) with a predicted MW of 66 413. RadD has been implicated in DNA repair following radiation or chemical damage (30,31). The peptide sequence of the RadD protein includes all seven of the motifs associated with a superfamily 2 (SF2) helicase, with the motifs aligning well to *E. coli* SF2 helicases RecG and RecQ. RadD also exhibits limited homology to the human XPB(ERCC3) protein. The protein binds to SSB and exhibits a DNA-independent ATPase activity that is stimulated by interaction with SSB (32). The structure of RadD has been determined (33). The protein contains all seven conserved SF2 motifs, with two RecA-like domains and a zinc finger. The protein appears to function as a monomer.

*In vivo*, deleting *radD* leads to an increase in crossovers and sensitivity to ionizing radiation. However, these phenotypes result in relatively minor effects on survival and RadD was overlooked for decades as work on bacterial recombination systems progressed. The importance of RadD becomes evident when *radD* deletions are introduced into cells lacking additional repair functions, particularly *uup* and *recG* (7,34). A *radD recG* double mutant is nearly incapable of growth and suppressors arise rapidly (7). The genetic results indicate that RecG and RadD operate in alternative pathways to deal with situations requiring the processing of stalled replication forks. One of these pathways, with RadD, also involves the RecA protein (7).

The structure of RadD (33) suggested that it may function as a helicase, and we have made extensive efforts to detect and characterize a helicase activity. However, to date, RadD helicase activity has not been observed on oligo-based helicase substrates of any structure under any conditions, despite the capacity of RadD to bind synthetic replication forks (34).

Here, we identify the molecular function of RadD. It is an accessory protein that accelerates RecA-mediated DNA strand exchange, with the novel trait that it is entirely RecA-dependent. Although on the surface, this function seems to overlap with that of RadA and RuvAB, these proteins and RadD are not interchangeable. In cells lacking RecG function, cells exhibit a severe growth defect in the absence of RadD even though both RadA and RuvABC are present and functional (7,34). In this study, we begin to explore the molecular basis of that effect.

## MATERIALS AND METHODS

### Protein purification

*E. coli* RecA and RadD proteins were purified in their native forms as described previously (17,32). The open reading frame of *E. coli* RadD K37R was PCR amplified and subcloned in-frame into NdeI/EcoRI digested pET21a resulting in plasmid pEAW755. STL2669(DE3) (exo1- ΔrecA derivative of AB1157 Tet^R^ (from Susan Lovett)) was transformed with pEAW755, and 10 l of cells were grown in LB containing 100 μg/ml Ampicillin at 37°C to an OD_600_ of 0.4. Expression of RadD K37R was induced by adding IPTG to a final concentration of 0.4 mM to each culture and growing cells for an additional 3 hrs at 37°C. Cells were pelleted, flash frozen and resuspended in 25% (w/v) Tris-sucrose solution to a 20% (w/v) cell pellet weight to volume ratio overnight. Three Roche Complete Protease Cocktail tablets were added and cells were lysed by sonication. Cell debris was pelleted, and supernatant was saved. RadD K37R was precipitated by adding ground ammonium sulfate to a final concentration of 0.176 g/ml and stirred gently at 4°C for 2 h. Precipitant mixture was pelleted and resuspended in R buffer (20 mM Tris-Cl, 0.1 mM EDTA, 1 mM DTT) plus 1 M ammonium sulfate and 10% glycerol. Protein was loaded onto a butyl sepharose column and eluted over a linear gradient of 10 column volumes from 1 M to 0 ammonium sulfate. RadD does not bind. Protein containing flow-through fractions were pooled and dialyzed three times against 2 l P buffer (20 mM potassium phosphate, 0.1 mM EDTA, 10% glycerol, 200 mM KCl) for 2 h each time. Dialyzed protein was loaded onto a ceramic hydroxyapatite column. RadD K37R eluted at around 15% of the way through a 10-column volume gradient against 1 M potassium phosphate, 10% glycerol, 1 mM DTT buffer. RadD K37R pools were dialyzed against R buffer containing 200 mM KCl and 10% glycerol 3 times and dialyzed fractions were loaded onto a Source 15S followed by a Source 15Q column where RadD eluted in the flow-through both times. Protein containing fractions were pooled and loaded onto a PBE94 column equilibrated with 2 column volumes R buffer containing 200 mM KCl and 10% glycerol. RadD K37R was eluted with a 10-column volume gradient against R + 1 M KCl, 10% glycerol, 1 mM DTT buffer. Peak fractions eluted approximately after 10% of the gradient was completed. RadD K37R containing fractions were pooled and tested for nuclease activity against φX 174 RFI, φX 174 virion and PstI digested φX 174 RFI DNA. Protein was then aliquoted, and flash frozen for −80°C storage. All proteins used in this work were greater than 98% pure and lacked detectable nuclease activities. The concentration of RadD and RadD K37R was determined using the measured extinction coefficient of 59 500 M^–1^ cm^–1^ (32). The concentration of the purified RecA protein was determined from the absorbance at 280 nm using the extinction coefficient of 2.23 × 10^4^ M^–1^ cm^–1^ (35).

### Strand exchange reactions

All reaction incubations were carried out at 37°C. 20 μM nt φX 174 Virion DNA (NEB # N3023S) was incubated with 1X RecA buffer (25 mM Tris-OAc (80% cation, pH 7.5), 1 mM dithiothreitol, 5% (w/v) glycerol, 3 mM potassium glutamate, and 10 mM magnesium acetate), 1 mM DTT, 2.5 mM phospho(enol)pyruvate (PEP), 10 U/ml pyruvate kinase. Unless otherwise indicated, 3.3 μM RecA was incubated for 10 min. 2.1 μM SSB and 3 mM ATP were added, and the reaction was incubated for another 10 min. Each reaction was initiated by the addition of 20 μM nt φX 174 RF1 DNA (NEB # N3022L) previously digested by PstI. 10 μl aliquots were taken at appropriate times and added to 5 μl of 3:2 6× Ficoll:10% SDS min and further incubated for 10 min. Ficoll:SDS was omitted from samples that were used for electron microscopy and instead immediately processed as described under electron microscopy and filamentation measurements. Gel samples were subjected to electrophoresis on a 0.8% TBE gel at 25 V for 16 h.

DNA gels were stained with ethidium bromide and bands for all double-stranded DNA species (joint molecules, nicked circular and ldsDNA) were quantified using ImageJ. Each experiment was repeated at least three times. Quantifications represent raw data unless otherwise indicated, with 100% of DNA representing the sum of the joint molecule, nicked circular and ldsDNA in each lane.

In addition to raw quantifications, quantification of intermediate resolution assays was normalized such that the % of dsDNA present as product nicked circles at times 0 and 30 min was set to 0 and 100%, respectively to allow more direct comparison of runs featuring the differing amounts of joint molecules present in three separate experiments that were initiated from three separate preparations of intermediate molecules. This plot and the raw data plots are both reported.

In typical reactions including RadD, RadD was added immediately (10 s or less) after the addition of linear dsDNA. In order-of-addition reactions, RadD was either added (i) with the addition of RecA, before the addition of SSB, ATP and ldsDNA, or (ii) after incubation of circular ssDNA with RecA, with the addition of SSB and ATP, before reaction initiation with ldsDNA.

### Intermediate isolation

Strand exchange reactions were scaled up to 400 μl, keeping the concentrations and ratios of all reagents otherwise the same as the standard conditions described above. Reactions were initiated as previously described. At peak intermediate formation (∼7–9 min), reactions were stopped by adding SDS to a final concentration of 0.2% and allowed to incubate for 15 min further at 37°C. Sepharose 2B-CL resin (2.5 ml) was equilibrated with 2B-CL Buffer (20 mM Tris-acetate, pH 7.4, 1 mM DTT, 0.65% glycerol, 11 mM magnesium acetate, 3 mM ammonium glutamate) with 5 column volumes. SDS-denatured proteins were separated from DNA molecules by loading stopped reactions manually onto the Sepharose 2B-CL resin bed drop by drop at room temperature. Gravity fractions were collected in two drop (∼100 μl) fractions and analyzed on a 0.8% 1× TAE agarose gel. Lanes containing the most concentrated DNA were pooled, quantified by comparison to standards on an agarose gel, stored at 4°C and used the same day.

### Intermediate resolution assay

Branch migration assays were carried out with 1× RecA buffer, 1mM DTT, 2.5 mM PEP, 10 units/ml pyruvate kinase, 3 mM ATP and 15 μM nucleotide DNA intermediates. Reactions were initiated by the addition of indicated amounts of RadD or RecA proteins. Aliquots (10 μl) were taken at the indicated times and incubated in a 3:2 6× Ficoll:SDS solution for 15 min at 37°C. Reactions were loaded onto a 0.8% 1× TBE gel and electrophoresed for 16 h at 25 V. DNA gels were stained with ethidium bromide, and bands for all double-stranded DNA species were quantified using ImageJ.

### ATP hydrolysis reactions

We utilized a spectrophotometric assay that has been described previously (17,36). In the reactions labeled, 10 μM nucleotides φX 174 Virion DNA was incubated with displayed amounts of RecA or RadD for 5 min with 1× RecA buffer 1mM dithiothreitol, 10 units/ml pyruvate kinase, 10 units/ml lactate dehydrogenase, 5 mM phospho(enol)pyruvate, and 2 mM NADH. Reactions were initiated by the addition of 5 μM ATP and 1 μM SSB protein. For strand exchange reactions 10 μM nucleotides φX 174 RF1 DNA was added 10 min after SSB and ATP addition. Readings were taken at 380 to monitor the conversion of NADH into NAD+ every 40 s.

### Electron microscopy and filament length measurements

A modified alcian method was used to visualize RecA nucleoprotein filaments. Activated grids were prepared as described previously (37). Samples for electron microscopy analysis were prepared as follows: reaction mixtures were prepared by pre-incubating RecA (6.7 μM), M13 circular ssDNA (20 μM), Tris-OAc (80% cation) buffer (25 mM), glycerol (5% (w/v)), potassium glutamate (3 mM), Mg (OAc)_2_ (10 mM) and DTT (1 mM) for 5 min. All reactions were carried out at 37°C without ATP regeneration system. The components were incubated for an additional 10 min with ATP (3 mM) and SSB (2.1 μM). RadD (0.5 μM) or compensating storage buffer was then added and incubation continued for another 2 min. ATPγS was then added to a final concentration of 10 mM and the samples were spread immediately. The reaction mixture was diluted to a final DNA concentration of 0.0004 μg/μl with ammonium acetate (200 mM), HEPES (10 mM; pH 7.5) and glycerol (10%), and adsorbed to an activated alcian grids for 3 min. The grids were then touched to a drop of the above buffer followed by floating on a drop of the same buffer for 1 min. The sample was then stained by touching to a drop of 5% uranyl acetate followed by floating on a fresh drop of 5% uranyl acetate for 30 s. Finally, the grid was washed by touching to a drop of double distilled water followed by immersion into double distilled water in two 10 ml beakers. After the samples were dried, they were rotary-shadowed with platinum. This protocol is designed for visualization of complete reaction mixtures and no attempt was made to remove unreacted material. Although this approach should yield results that provide insight into reaction components, it does lead to samples with a high background of unreacted proteins.

A molecule was considered gapped if it had a detectable region of SSB-coated DNA of any size. The observed (by visual judgement) length of the RecA filaments and the length of SSB-coated DNA were used to assign molecules to five categories: full filaments, medium filaments, small filaments, very small filaments or SSB/DNA molecules. Linearized DNA molecules likely originating from shearing force during pipetting were also considered. The filaments are considered ‘Full filaments’ when the circular DNA molecule is completely encompassed by RecA or when they had small discontinuities in the regular striated pattern. Medium filaments were smaller in length than full filaments, but still had substantial regions of nucleoprotein filament. Small filaments are less than half the length of full filaments, and often had regions of obvious SSB binding. Very small filamented molecules are those with just detectable segments of RecA filamented regions, with the rest of the molecule coated with SSB. SSB coated DNA molecules were the ones that are bound only by the SSB protein.

Imaging and photography were carried out with a TECNAI G2 12 Twin Electron Microscope (FEI Co.) equipped with a 4k × 4k Gatan Ultrascan CCD camera. Digital images of the nucleoprotein filaments were taken at × 15000 magnification.

At least fifty circular DNA molecules coated with RecA were measured from samples with and without RadD. Samples with RecA (without RadD) contained mostly large circular filaments while samples with both RecA and RadD showed a broader size range (see Results). Each molecule was measured three times using Metamorph analysis software and the average length was calculated in nm. The 500 nm scale bar was used as a standard to calculate the number of pixels/μm. Plots were generated using GraphPad Prism Software.

All experiments presented in this work were carried out at least three times with consistent results.

## RESULTS

The model reaction shown in Figure 1A serves to illustrate the steps of a RecA reaction. The RecA protein forms helical filaments on the single-stranded DNA circle, pairs the bound DNA with homologous sequences in a linear duplex DNA to create a joint molecule, and then promotes a DNA strand exchange reaction that is coupled to ATP hydrolysis (15,38). Once the DNA is paired, strand exchange of the substrate DNAs (derived from φX 174 bacteriophage and 5386 bp in length) requires multiple minutes to complete.

In the experiments shown in Figure 1, panels B and C, the RecA protein is present at a concentration (3 μM), just less than half that required to saturate available ssDNA binding sites (20 μM ssDNA in total nucleotides would provide 6.7 μM in RecA binding sites, with three nucleotides per binding site). The 20 μM ssDNA in nucleotides translates to 3.7 nM φX174 ssDNA molecules. The concentration of dsDNA in nucleotides (20 μM dsDNA in nucleotides gives 10 μM in base pairs) corresponds to 1.86 nM molecules. Significant nicked circular DNA product is evident at the 10 min timepoint. When 3.7 nM RadD was added to the reaction at the same time as the linear duplex substrate, at a concentration about twice that of the concentration of the linear dsDNA molecules, products appeared much faster, within 2 min. Addition of a RadD mutant lacking ATPase activity, RadD K37R (32), has no substantial effect on the reaction in this experiment, indicating that the RadD ATPase activity was important. A small effect of RadD K37R may be present, an issue that requires further future assessment.

To continue this experiment, we investigated the effect of RadD on the strand exchange reaction when a higher, stoichiometric concentration of RecA protein (Figure 1 D, E) was present. Strand exchange products appeared earlier under these conditions. However, the addition of wild type RadD protein still accelerated the reaction substantially.

We note that the reactions with RadD in Figure 1 proceed to greater than 50% completion, as do the reactions with RecA alone. The final yield of nicked circular products is similar in reactions with and without RadD. This indicates that RadD is accelerating the DNA strand exchange reaction in the same direction in which it is generally promoted by RecA protein. There is no indication of strand exchange reversal. Hence, RadD does not appear to be an anti-recombinase and there is no indication in any experiment that it promotes strand exchange reversal. RadD is a RecA accessory that complements RecA function.

**Figure 2.**
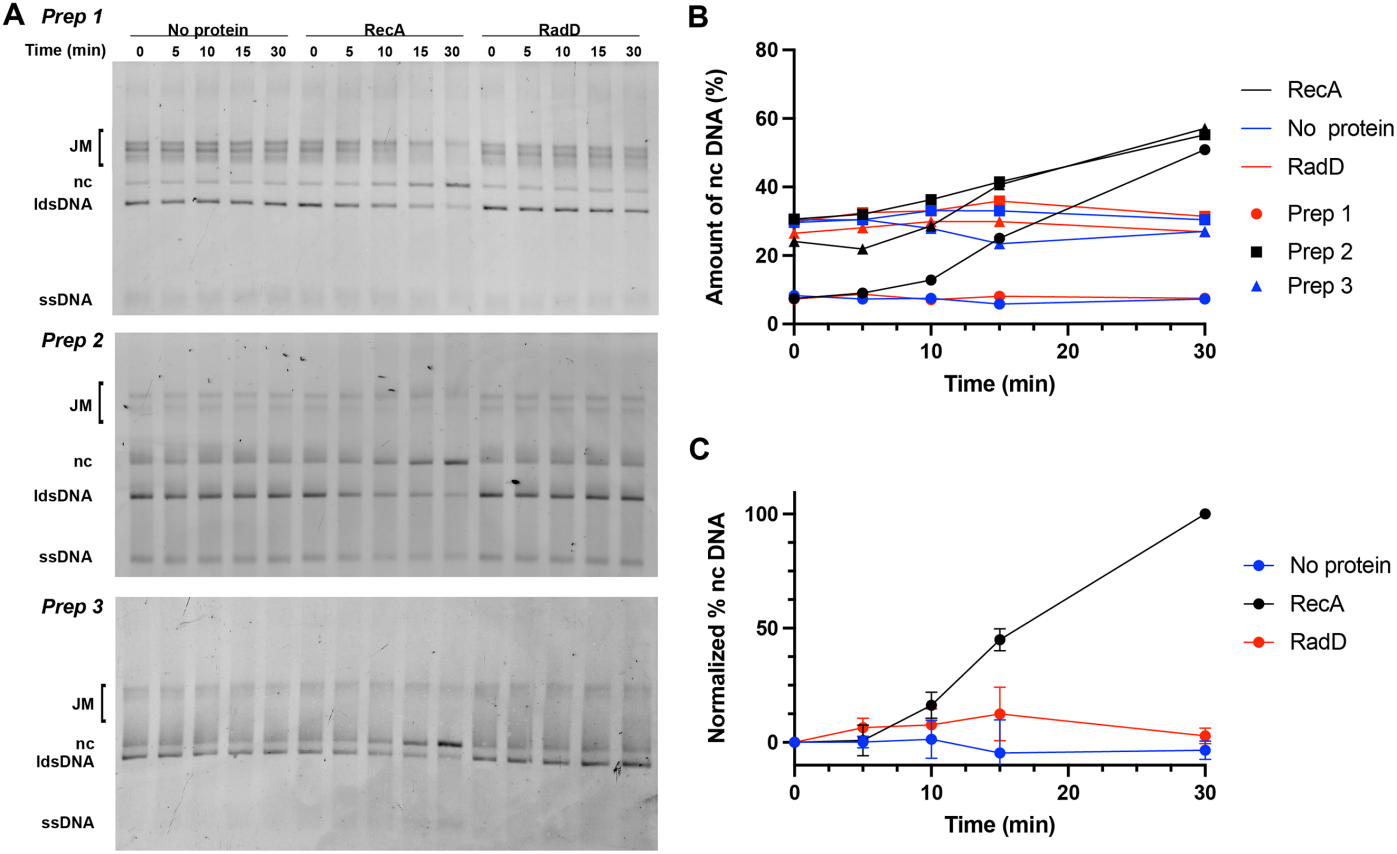
RadD function is RecA-dependent. (**A**) Isolated protein-free strand exchange intermediates were incubated alone, with RecA (3 μM), or RadD (3.7 nM). Results from experiments prepared from three independent preparations of joint molecules (JM) are shown. (**B**) Raw quantifications of nc DNA from three independent experiments started from three separate preparations of JMs. (**C**) Quantifications of nc DNA from all three gels shown in (A), averaged, with results normalized such that the start and end points of the RecA reaction are set at 0 and 100, respectively.

The effect of RadD is entirely dependent on RecA protein. In the experiment of Figure 2, DNA strand exchange was initiated by RecA protein as described in Methods, under the conditions of Figure 1B. After 7–9 min, DNA strand exchange intermediates were carefully isolated and introduced into a new reaction mixture. When RecA was added back to these intermediates, completion of strand exchange was evident. The addition of RadD protein alone to the intermediates had no effect on the DNA in spite of the presence of abundant joint molecules with DNA branch points. Thus, the effects of RadD are completely RecA-dependent.

**Figure 3.**
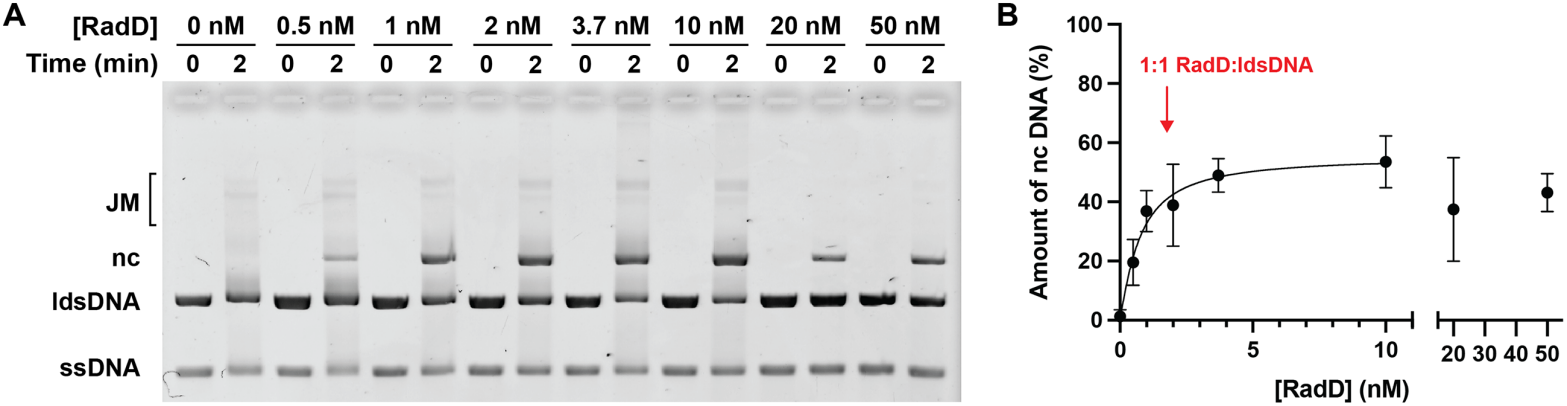
Stimulation of RecA mediated strand exchange by RadD; effects of concentration. Strand exchange reactions with RadD titrated from 1 to 50 nM. (**A**) Sample reaction with RadD titration. Reactions were carried out under the conditions of Figure 1D (with sufficient RecA to saturate available ssDNA binding sites), except that the RadD concentration was varied and reactions were stopped after 2 min. (**B**) Quantification of three experiments identical to that in panel A. Arrow indicates the concentration of RadD that would be equivalent to the concentration of linear dsDNA molecules and thus the maximum concentration of RecA-generated branched intermediates in the experiment.

The concentration of single-stranded φX174 DNA in the standard reaction mixture, 20 μM in nucleotides, translates to 3.7 nM in molecules. The concentration of linear dsDNA, also 20 μM in nucleotides, is only 10 μM in base pairs and is present at 1.85 nM, only half the concentration of ssDNA when measured in molecules. The reactions in Figure 1B, C utilize 3 μM RecA and 3.7 nM RadD proteins, indicating that near stoichiometric amounts of RadD (relative to active RecA filaments and/or strand exchange branch points) are completely adequate to observe an optimal reaction. This concept is reinforced in Figure 3, where the RadD concentration was varied from 1 to 50 nM under otherwise standard reaction conditions. The effect of RadD was apparent at both low and high concentrations. The production of products after 2 min is used as a rough indicator of reaction rate. With this benchmark, product formation saturates at or near the concentration of RadD that represents the approximate concentration of RecA-generated DNA branch points undergoing DNA strand exchange. Although more detailed explorations of reaction rates are needed to pinpoint interaction stoichiometries, the results suggest that as few as a single RadD monomer is sufficient to accelerate DNA strand exchange mediated by a particular RecA filament.

**Figure 4.**
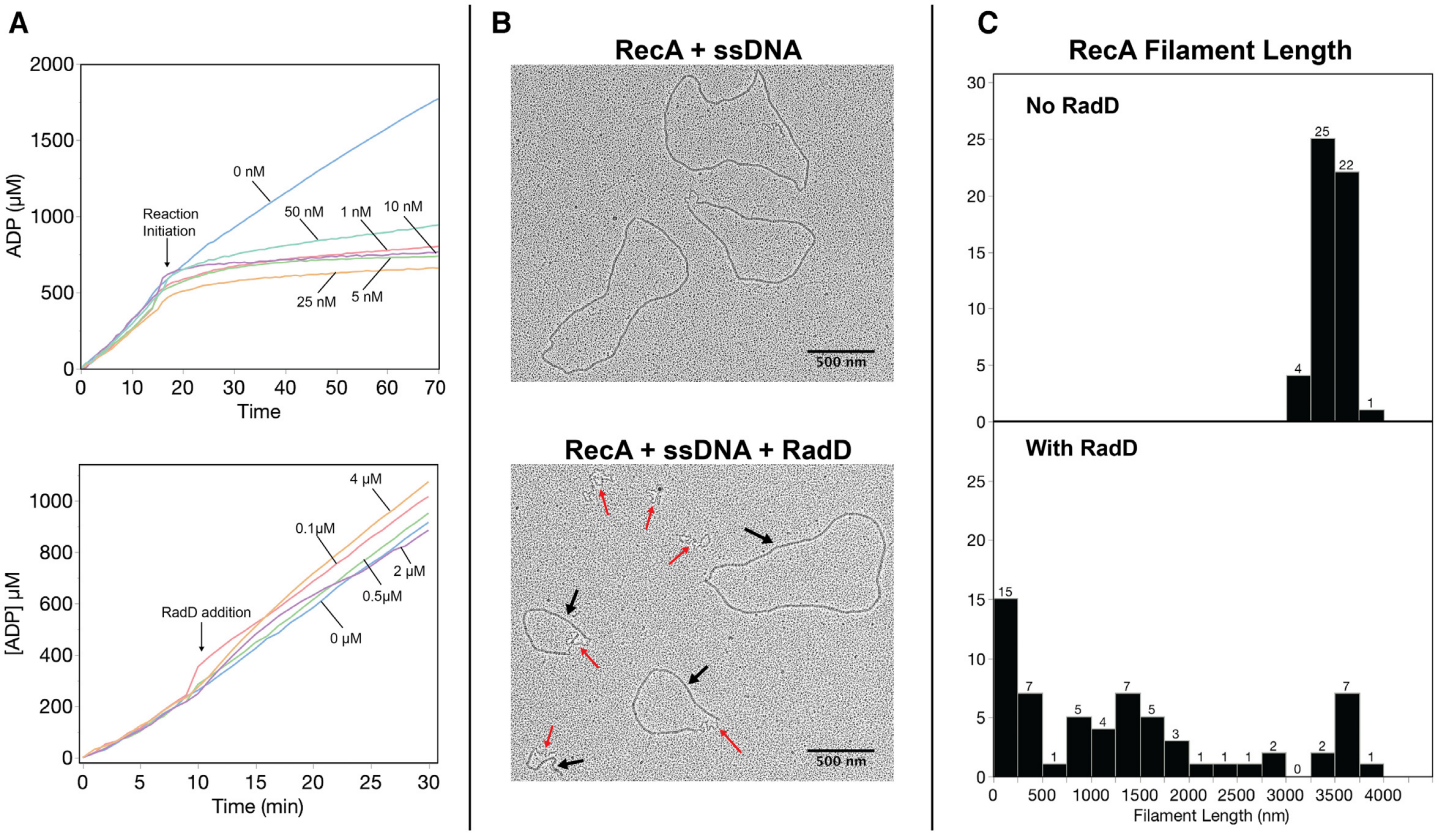
RecA protein filaments are disassembled by RadD. (**A**) Top panel – ATP hydrolysis profiles of RecA during strand exchange without and with varying concentrations of RadD. Reactions were initiated by the addition of duplex DNA at 15 min. Where RadD is present, it was added at the concentrations indicated and at the same time as the duplex DNA. Bottom panel – RecA ATP hydrolysis trace on single stranded DNA in the presence of various RadD K37R concentrations as indicated. RadD was added at 10 min after the reaction was initiated at 37°C. B. Representative electron microscopy images of RecA filaments on single stranded DNA without RadD (top) and with RadD (bottom). Black arrows indicate regions of circular ssDNA coated with RecA. Red arrows indicate regions of ssDNA coated with SSB. (**C**) Quantification of observed RecA filament lengths. RadD K37R titration into RecA ATP hydrolysis reactions after 10 min of RecA incubation with DNA. Numbers reflect the total number of filament lengths that fell into each size bin. For reactions without and with RadD, *n* = 52 and 62, respectively.

What happens to RecA filaments when RadD is added to a reaction? A few observations are presented in Figure 4 that suggest that RadD has a potent and destructive effect on RecA filaments. We first followed the RecA and RadD ATPase activities during the DNA strand exchange reaction, adding RadD protein at the same time as we added linear duplex DNA to initiate the reaction. We note that levels of RadD ATPase would be minimal in this trial at the very low RadD concentrations used (1–50 nM). When duplex DNA was added to the reaction in the absence of RadD (Figure 4A, upper panel, 0 nM), the rate of ATP hydrolysis declined by about 20% in accordance with previous observations, reflecting a shift in RecA filament state that reflects duplex substrate alignment (17). However, the addition of RadD along with the dsDNA produced a very rapid and much greater drop in ATP hydrolysis rates (Figure 4A, top panel). This occurred even when RadD was present at only 1 nM in concentration. This suggested that the RadD-mediated enhancement of RecA-mediated strand exchange involved the elimination or alteration of the RecA filaments. To explore this further in a simpler system, we examined the effect of RadD on RecA filaments formed on circular ssDNA (with no strand exchange occurring). In this case, the addition of RadD again produced a sharp drop in ATP hydrolysis (data not shown). The addition of RadD K37R at any concentration had no effect on the ongoing ATPase reaction of RecA (Figure 4A, lower panel). We further explored the effect of wild type RadD on these RecA filaments by electron microscopy. Within 2 min after the addition of RadD protein, full sized RecA filaments were much reduced, replaced by much shorter fragments (Figure 4B and C). The results indicate that RadD facilitates RecA filament disassembly as well as an acceleration of the RecA-mediated strand exchange reaction.

**Figure 5.**
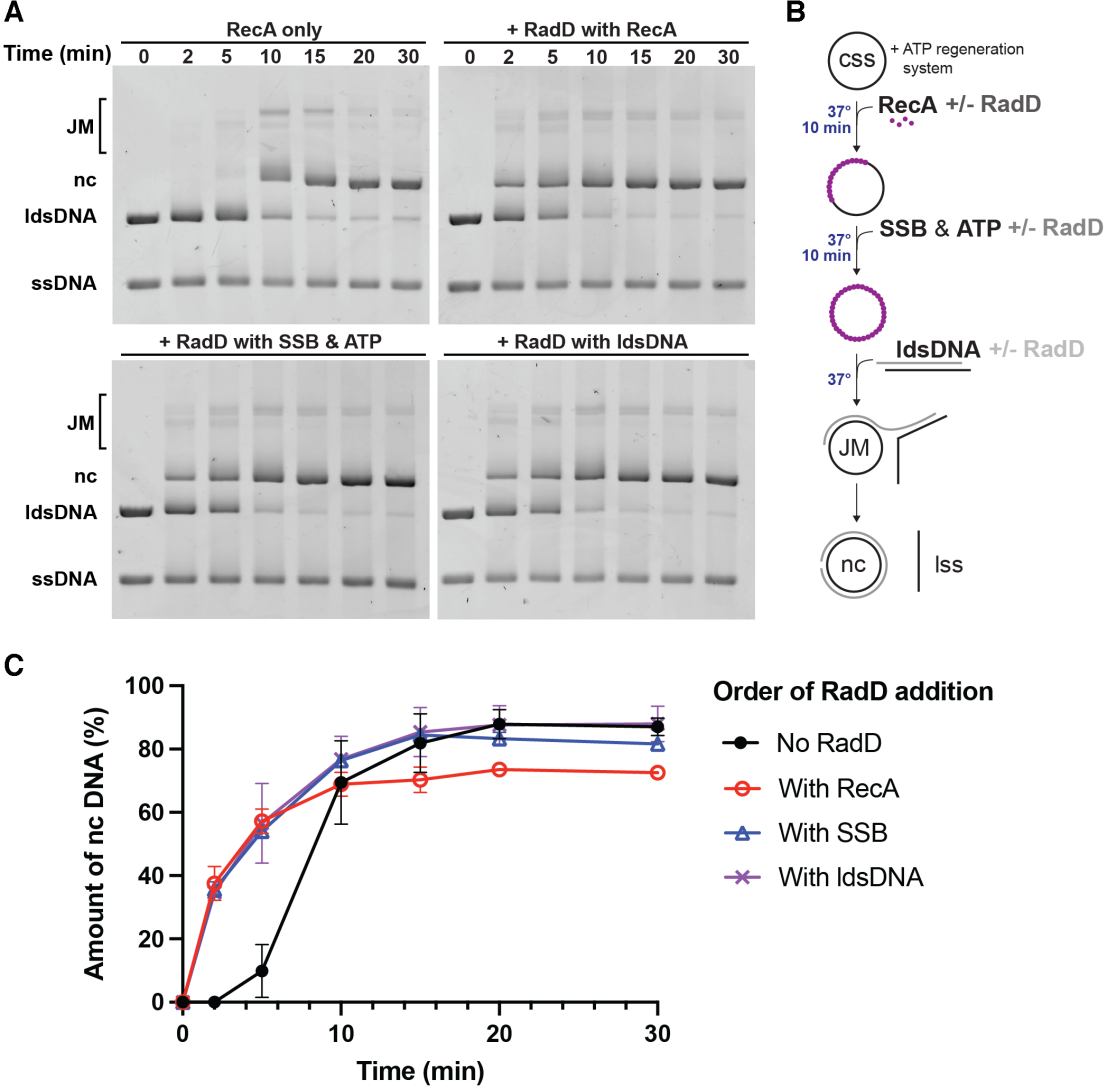
Effects of altering order of addition. RadD stimulates strand exchange when added to the reaction at different pre-incubation steps. Reaction conditions were identical to those used in Figure 1B, with 20 μM nt cssDNA and ldsDNA, 3 μM RecA, and 3.7 nM RadD. (**A**) Strand exchange time courses of reactions where RadD is excluded, added with RecA, added with SSB and ATP, or added with ldsDNA (at time zero). (**B**) Diagram of strand exchange reaction incubations and order of addition of reagents. The reactions are initiated by the addition of ldsDNA. (**C**) Quantifications of nc DNA from three independent sets of order of addition experiments with the average value plotted and standard deviations represented as error bars.

Can RadD-mediated disassembly of RecA filaments inhibit DNA strand exchange if RadD is added too early? In Figure 5, we explore the effects of RadD order of addition. Under the conditions of Figure 1B, RadD was added either with the RecA protein (after ssDNA but prior to ATP and SSB), with the ATP and SSB but prior to the dsDNA, and with the dsDNA as in Figure 1B. The results were essentially identical in all experiments, with RadD stimulating the strand exchange reactions to the same extent. Assuming that RadD is promoting RecA disassembly while accelerating DNA strand exchange, addition of RadD prior to the onset of strand exchange does not appear to affect the overall reaction. RecA protein can initiate DNA strand exchange with short RecA filaments and RadD can apparently accelerate strand exchange whether RecA is otherwise coating the entire ssDNA or not.

## DISCUSSION

The major conclusion of this work is that the formerly enigmatic RadD protein is a RecA-dependent accessory protein, accelerating the DNA strand exchange reactions promoted by RecA protein. RadD binds to DNA and hydrolyzes ATP independently of any other protein (31). However, RadD exhibits no independent helicase activity *in vitro* and it will not initiate DNA pairing or strand exchange. RadD will not act even on strand exchange intermediates created by RecA if the RecA protein is first removed (Figure 2). A helicase or DNA translocase function that is somehow activated by RecA protein represents a likely functional scenario. RadD acts at very low concentrations (Figure 3), near or equivalent to the concentration of RecA-created DNA branch points. Its addition results in significant RecA filament disassembly (Figure 4).

As already noted, there are other proteins that can process branched DNA intermediates created by RecA protein, including RadA and RuvAB. The RadD activity is not equivalent to RadA or RuvAB. Neither RadA nor RuvAB will substitute for RadD *in vivo*. The presence of active RadA and RuvB in the same cells in no way alleviates the growth defect seen in the *ΔradDΔrecG* double mutants (7). In addition, that same severe growth defect is suppressed if RecA expression is reduced or RecA protein loading by RecFOR is eliminated (7). These genetic observations, coupled to the RecA-dependence of the RadD activity, indicate a uniquely close association between RecA and RadD, one in which an intermediate produced by RecA is toxic unless processed by RadD in these cells. RuvB and RadA, at least on their own, do not provide alternative paths.

Among proteins that process strand exchange intermediates, RadD is so far unique in its complete dependence on the presence of RecA. RadD affects RecA-mediated strand exchange approximately the same way regardless of when it is added to the reaction. This suggests that the process is sequential. RecA promotes DNA pairing. A relatively short RecA filament may be sufficient for this step. RadD then promotes more rapid movement of the branched DNA structure that has been created by RecA.

In principle, RadD could simply accelerate the RecA-mediated process of strand exchange, acting on RecA filaments in some manner. The substrate for RadD, rather than being some particular DNA structure, could be the RecA nucleoprotein filament and DNAs that are bound to it. Alternatively, RadD could have a cryptic helicase activity that is activated by interaction with RecA. RadD does not block the reaction if added prior to RecA. However, RadD does have the effect of promoting RecA filament dissociation even when the RecA filament is not promoting strand exchange. Shortened RecA filaments, if they are long enough to initiate strand exchange, appear to be sufficient to trigger rapid strand exchange in the presence of RadD. We note that unlike RuvAB and RadA, the reactions promoted by RadD accelerate strand exchange in only one direction, the same direction as the reaction proceeds with RecA alone. Product formation in many reactions well exceeds 50%. There is no evidence in the data so far that a reversal of RecA-mediated strand exchange can be catalyzed by RadD. The data thus favor the notion that RadD is an accessory, a complement to RecA rather than an anti-recombinase.

Mechanistically, RadD appears to require as few as 1 monomer per RecA-generated DNA branch point. The RadD ATPase activity is clearly important, although a possible small effect of RadD K37R (see Figure 1 and its description in Results) requires further assessment. The activity of RadD is entirely dependent on RecA. We can imagine at least three functional scenarios. (i) RadD could function only in the presence of and interacting with an active RecA nucleoprotein filament, perhaps promoting rapid strand exchange while dissociating the RecA ahead of it. (ii) RadD could be a RecA-activated helicase, acting on branch points while dissociating RecA but not requiring further RecA interaction once activated. (iii) RadD could require addition of a RecA subunit for activity, much like DNA polymerase V secures a RecA subunit prior to activation of its translesion DNA synthesis function (39–41). This list is not exhaustive and the proposals in it may not be mutually exclusive. These and numerous additional mechanistic questions await resolution with further experimentation.

During exponential growth of oxygenated cells in a rich media, DNA repair is required often and the time window in which it must occur is relatively limited. Our understanding of recombinational DNA repair and other processes has not always considered the question: how does DNA repair support replication in a timely fashion, restoring the DNA templates fast enough to be ready for a new replication fork following as quickly as 15–20 min behind? Recombinational DNA repair requires the successive steps of RecA-loading by other proteins, RecA-mediated strand exchange, and then processing of the structures created by RecA. The relatively slow rate of RecA-mediated strand exchange, which can migrate DNA branches through thousands of bp but on a timescale of minutes, has always made it difficult to envision catalytic competence with respect to the cell cycle, although this problem has been little discussed.

As the constellation of proteins involved in recombinational DNA repair continues to expand, the apparent complexity of the possible pathways increases and questions multiply. What protein acts when, and in what DNA structural context? In spite of decades of work on these processes, our understanding remains inchoate. The rescue of replication forks and the repair of post-replication gaps and double strand breaks is clearly not limited temporally by the known catalytic properties of the RecA protein. There are many other proteins that augment and regulate RecA, with RadD being a particularly specialized example. The processing of intermediates generated by RecA protein must involve RadD in some important cellular contexts, as the lack of processing is toxic (7). RecG may deal with the toxicity of these intermediates in a different way – by reversing them (27,28). The growth defect seen in cells lacking both RadD and RecG function (7) suggests that RadD and RecG may represent opposing ways to deal with toxic strand exchange intermediates created by RecA—extending the exchanged region rapidly with RadD or reversing the intermediate with RecG.

Although neither RadA nor RuvB will substitute for RadD, the genetics do not eliminate the possibility that RadD may in some manner act in concert with one or both of these proteins. We note that a *radArecG* double mutant exhibits a strongly synergistic sensitivity to DNA damaging agents, albeit without the severe growth defect of the *radDrecG* combination (42,43).

We do not yet know how the RadD protein interfaces with RecA or under what precise DNA metabolism circumstances it may act. The circumstances under which RadA or RuvB may operate are similarly unclear. The current study suggests many avenues for further investigation of these and many other questions. The work also provides a path to identification of similar recombinase-dependent functions in eukaryotes.

## DATA AVAILABILITY

All data underlying this study is included within the article itself.
